# An inclusive Research Education Community (iREC) Model to Facilitate Undergraduate Science Education Reform

**DOI:** 10.3389/feduc.2024.1442318

**Published:** 2024-08-20

**Authors:** Denise L. Monti, Julia C. Gill, Tamarah L. Adair, Sandra D. Adams, Yesmi Patricia Ahumada-Santos, Isabel Amaya, Kirk R. Anders, Justin R. Anderson, Mauricio S. Antunes, Mary A. Ayuk, Frederick N. Baliraine, Tonya C. Bates, Andrea R. Beyer, Suparna S. Bhalla, Tejas Bouklas, Sharon K. Bullock, Kristen A. Butela, Christine A. Byrum, Steven M. Caruso, Rebecca A. Chong, Hui-Min Chung, Stephanie B. Conant, Brett M. Condon, Katie E. Crump, Tom D’Elia, Megan K. Dennis, Linda C. DeVeaux, Lautaro Diacovich, Arturo Diaz, Iain Duffy, Dustin C. Edwards, Patricia C. Fallest-Strobl, Ann M. Findley, Matthew R. Fisher, Marie P. Fogarty, Victoria J. Frost, Maria D. Gainey, Courtney S. Galle, Bryan Gibb, Urszula P. Golebiewska, Hugo C. Gramajo, Anna S. Grinath, Jennifer A. Guerrero, Nancy A. Guild, Kathryn E. Gunn, Susan M. Gurney, Lee E. Hughes, Pradeepa Jayachandran, Kristen C. Johnson, Allison A. Johnson, Alison E. Kanak, Michelle L. Kanther, Rodney A. King, Kathryn P. Kohl, Julia Y. Lee-Soety, Lynn O. Lewis, Heather M. Lindberg, Jaclyn A. Madden, Breonna J. Martin, Matthew D. Mastropaolo, Sean P. McClory, Evan C. Merkhofer, Julie A. Merkle, Jon C. Mitchell, María Alejandra Mussi, Fernando E. Nieto-Fernandez, Jillian C. Nissen, Imade Y. Nsa, Mary G. O’Donnell, R. Deborah Overath, Shallee T. Page, Andrea Panagakis, Jesús Ricardo Parra Unda, Michelle B. Pass, Tiara G. Perez Morales, Nick T. Peters, Ruth Plymale, Richard S. Pollenz, Nathan S. Reyna, Claire A. Rinehart, Jessica M. Rocheleau, John S. Rombold, Ombeline Rossier, Adam D. Rudner, Elizabeth E. Rueschhoff, Christopher D. Shaffer, Mary Ann V. Smith, Amy B. Sprenkle, C. Nicole Sunnen, Michael A. Thomas, Michelle M. Tigges, Deborah M. Tobiason, Sara S. Tolsma, Julie Torruellas Garcia, Peter Uetz, Edwin Vazquez, Catherine M. Ward, Vassie C. Ware, Jacqueline M. Washington, Matthew J. Waterman, Daniel E. Westholm, Keith A. Wheaton, Simon J. White, beth C. Williams, Daniel C. Williams, Ellen M. Wisner, William H. Biederman, Steven G. Cresawn, Danielle M. Heller, Deborah Jacobs-Sera, Daniel A. Russell, Graham F Hatfull, David J. Asai, David I. Hanauer, Mark J. Graham, Viknesh Sivanathan

**Affiliations:** 1 Hicks Honors College, University of North Florida, Jacksonville, Florida 32224, USA; 2 Metropolitan Policy Department, Brookings Institution, Washington, District of Columbia 20036, USA; 3 Department of Biology, Baylor University, Waco, Texas 76798, USA; 4 Biology Department, Montclair State University, Montclair, New Jersey 07043, USA; 5 Facultad de Ciencias Químico Biológicas, Universidad Autónoma de Sinaloa, Culiacán, Sinaloa 80030, Mexico; 6 Department of Cell and Developmental Biology, University of Michigan Medical School, Ann Arbor, Michigan 48109, USA; 7 Biology Department, Gonzaga University, Spokane, Washington 99258, USA; 8 Department of Biological Sciences, Southeastern Louisiana University, Hammond, Louisiana 70402, USA; 9 Department of Biological Sciences, University of North Texas, Denton, Texas 76203, USA; 10 Department of Biology, Howard University, Washington, District of Columbia 20059, USA; 11 Biology & Kinesiology, LeTourneau University, Longview, Texas 75602, USA; 12 Department of Biological Sciences, University of North Carolina at Charlotte, Charlotte, North Carolina 28223, USA; 13 Department of Biology, Virginia State University, Petersburg, Virginia 23806, USA; 14 Natural Science, Mount Saint Mary College, Newburgh, New York 12550, USA; 15 Microbiology, Immunology, and Molecular Genetics, University of California Los Angeles, Los Angeles, California 90095, USA; 16 Department of Biological Sciences, University of Pittsburgh, Pittsburgh, Pennsylvania 15260, USA; 17 Department of Biology, College of Charleston, Charleston, South Carolina 29424, USA; 18 Department of Biological Sciences, University of Maryland, Baltimore County, Baltimore, Maryland 21250, USA; 19 School of Life Sciences, University of Hawaii at Manoa, Honolulu, Hawaii 96822, USA; 20 Biology, University of West Florida, Pensacola, Florida 32514, USA; 21 Department of Biology, University of Detroit Mercy, Detroit, Michigan 48221, USA; 22 Biomedical Sciences, Rocky Vista University - Montana College of Osteopathic Medicine, Billings, Montana 59106, USA; 23 Department of Biological Sciences, Nova Southeastern University, Fort Lauderdale, Florida 33314, USA; 24 Department of Biology, Indian River State College, Ft. Pierce, Florida 34981, USA; 25 Biology, Marist College, Poughkeepsie, New York 12601, USA; 26 Biology Department, New Mexico Institute of Mining and Technology, Socorro, New Mexico 87801, USA; 27 Departamento de Microbiología Básica, Facultad de Ciencias Bioquímicas y Farmacéuticas-UNR, Rosario, Santa Fe 2000, Argentina; 28 Department of Biology, La Sierra University, Riverside, California 92505, USA; 29 Department of Natural Science, Saint Leo University, Saint Leo, Florida 33574, USA; 30 Biological Sciences, Tarleton State University, Stephenville, Texas 76402, USA; 31 Mathematics and Sciences, Neumann University, Aston, Pennsylvania 19014, USA; 32 Biology - School of Sciences, University of Louisiana at Monroe, Monroe, Louisiana 71209, USA; 33 Biology Department, Oregon Coast Community College, Newport, Oregon 97366, USA; 34 Science and Math, Durham Tech, Durham, North Carolina 27703, USA; 35 Biology, Winthrop University, Rock Hill, South Carolina 29733, USA; 36 Chemistry and Physics, Western Carolina University, Cullowhee, North Carolina 28723, USA; 37 Biology, South Texas College, McAllen, Texas 78501, USA; 38 Biological and Chemical Sciences, New York Institute of Technology, Old Westbury, New York 11568, USA; 39 Biological Sciences and Geology, Queensborough Community College, Bayside, New York 11364, USA; 40 Department of Biological Sciences, Idaho State University, Pocatello, Idaho 83201, USA; 41 Integrative Biology, University of Texas at San Antonio, San Antonio, Texas 78249, USA; 42 Molecular Cellular and Developmental Biology, University of Colorado, Boulder, Boulder, Colorado 80309, USA; 43 Science, Mount St. Mary’s University, Emmitsburg, Maryland 21727, USA; 44 School of Biology, University of St Andrews, St Andrews, Fife KY16 9ST, Scotland; 45 Basic and Clinical Sciences, Albany College of Pharmacy and Health Sciences, Albany, New York 12208, USA; 46 Department of Life Sciences, University of New Hampshire Manchester, Manchester, New Hampsire 03101, USA; 47 Center for Biological Data Science, Virginia Commonwealth University, Richmond, Virginia 23238, USA; 48 Department of Biology, University of North Georgia, Dahlonega, Georgia 30597, USA; 49 Biology, Bryn Mawr College, Bryn Mawr, Pennsylvania 19010, USA; 50 Biology, Western Kentucky University, Bowling Green, Kentucky 42101, USA; 51 Department of Biology, Saint Joseph’s University, Philadelphia, Pennsylvania 19131, USA; 52 Dept. of Biological Sciences, University of Mary Washington, Fredericksburg, Virginia 22401, USA; 53 Biology, Virginia Western Community College, Roanoke, Virginia 24015, USA; 54 Life Sciences Department, Harford Community College, Bel Air, Maryland 21015, USA; 55 Integrated Science, Business and Technology, La Salle University, Philadelphia, Pennsylvania 19141, USA; 56 Department of Biology, University of Evansville, Evansville, Indiana 47722, USA; 57 Science and Mathematics, Northern State University, Aberdeen, South Dakota 57401, USA; 58 Biological Sciences, SUNY Old Westbury, Old Westbury, New York 11568, USA; 59 Microbiology, University of Lagos, Lagos, Lagos 101017, Nigeria; 60 Department of Biology, Thiel College, Greenville, Pennsylvania 16125, USA; 61 Mathematics and Sciences, Texas Southmost College, Brownsville, Texas 78520, USA; 62 College of Health and Natural Sciences, Franklin Pierce University, Rindge, New Hampshire 03461, USA; 63 STEM Academy, Salish Kootenai College, Pablo, Montana 59855, USA; 64 Biological Sciences, Benedictine University, Lisle, Illinois 60532, USA; 65 Plant Pathology, Entomology, and Microbiology, Iowa State University, Ames, Iowa 50011, USA; 66 Department of Biology, Ouachita Baptist University, Arkadelphia, Arkansas 71998, USA; 67 Molecular Biosciences, University of South Florida, Tampa, Florida 33620, USA; 68 Biology, University of Massachusetts, Amherst, Amherst, Massachusetts 01003, USA; 69 Salish Sea Research Institute, Northwest Indian College, Bellingham, Washington 98226, USA; 70 University Paris-Saclay, CEA, CNRS, Institute for Integrative Biology of the Cell (I2BC), Gif-sur-Yvette 91190, France; 71 Ottawa Institute of Systems Biology and Department of Biochemistry, Microbiology and Immunology, University of Ottawa, Ottawa, Ontario K1S2Y8, Canada; 72 School of Natural Sciences, Indiana University Southeast, New Albany, Indiana 47150, USA; 73 Department of Biology, Washington University in St. Louis, St. Louis, Montana 63105, USA; 74 Biology, Penn State Schuylkill, Schuylkill Haven, Pennsylvania 17972, USA; 75 Biology, Salem State University, Salem, Massachusetts 01970, USA; 76 Chemistry and Biochemistry, Minnesota State University Moorhead, Moorhead, Minnesota 56563, USA; 77 Biology, Carthage College, Kenosha, Wisconsin 53140, USA; 78 Department of Biology, Northwestern College, Orange City, Iowa 51041, USA; 79 Biology Department, University of Puerto Rico at Cayey, Cayey, Puerto Rico 00736, USA; 80 Biological Sciences, Lehigh University, Bethlehem, Pennsylvania 18015, USA; 81 Department of Biology and Chemistry, Alliance University, New York, New York 10004, USA; 82 Biology Department, Eastern Nazarene College, Quincy, Massachusetts 02170, USA; 83 Biology, The College of St. Scholastica, Duluth, Minnesota 55811, USA; 84 Biochemistry, Microbiology & Immunology, University of Ottawa, Ottawa, Ontario K1G 5Z3, Canada; 85 Molecular and Cell Biology, University of Connecticut, Storrs, Connecticut 06268, USA; 86 Translational and Molecular Medicine, University of Ottawa, Ottawa, Ontario K1H 8M5, Canada; 87 Department of Biology, Coastal Carolina University, Conway, South Carolina 29528, USA; 88 Center for the Advancement of Science Leadership and Culture, Howard Hughes Medical Institute, Chevy Chase, Maryland 20815, USA; 89 Department of Biology, James Madison University, Harrisonburg, Virginia 22807, USA; 90 Department of English, Indiana University of Pennsylvania, Indiana, Pennsylvania 15705, USA; 91 STEM Program Evaluation and Research Lab (STEM-PERL), Department of Ecology and Evolutionary Biology, Yale University, New Haven, Connecticut 06511, USA

## Abstract

Over the last two decades, there have been numerous initiatives to improve undergraduate student outcomes in STEM. One model for scalable reform is the inclusive Research Education Community (iREC). In an iREC, STEM faculty from colleges and universities across the nation are supported to adopt and sustainably implement course-based research – a form of science pedagogy that enhances student learning and persistence in science. In this study, we used pathway modelling to develop a qualitative description that explicates the HHMI Science Education Alliance (SEA) iREC as a model for facilitating the successful adoption and continued advancement of new curricular content and pedagogy. In particular, outcomes that faculty realize through their participation in the SEA iREC were identified, organized by time, and functionally linked. The resulting pathway model was then revised and refined based on several rounds of feedback from over 100 faculty members in the SEA iREC who participated in the study. Our results show that in an iREC, STEM faculty organized as a long-standing community of practice leverage one another, outside expertise, and data to adopt, implement, and iteratively advance their pedagogy. The opportunity to collaborate in this manner and, additionally, to be recognized for pedagogical contributions sustainably engages STEM faculty in the advancement of their pedagogy. Here, we present a detailed pathway model of SEA that, together with underpinning features of an iREC identified in this study, offers a framework to facilitate transformations in undergraduate science education.

## Introduction

Longstanding calls for transformation in undergraduate science education highlight the continued rate at which students are leaving the sciences for other disciplines, as well as the high number of students not being prepared to enter modern STEM careers (PCAST, 2012; Brewer & Smith, 2011). Consequently, and for nearly two decades now, there has been significant investment in initiatives of various forms to support STEM faculty in the adoption and implementation of updated curricular content and evidence-based teaching practices in the undergraduate science classroom (Pfund et al., 2009; Vasaly et al., 2014; Smith, 2015). Despite a large cross-section of the STEM community coalescing around the curricula and pedagogical changes necessary for transformation (National Research Council, 2003; Brewer & Smith, 2011; Graham et al., 2013), there remains a shortfall of large-scale adoption and sustained implementation (Stains et al., 2018). To accelerate the pace of national change, there is a need to identify and leverage strategies that have proven to be effective in facilitating pedagogical transformation (Smith, 2018; Smith, 2019).

One model that has shown success in facilitating reform at scale is the *inclusive Research and Education Community*, or iREC (Hanauer et al., 2017). An iREC occurs when STEM faculty from institutions of higher education are supported to adopt and implement course-based research, collaboratively. Course-based research is a discovery-based approach to teaching science that exemplifies evidence-based teaching practices, transforms the undergraduate laboratory course experience, and improves student learning and their persistence in science (Auchincloss et al., 2014; Graham et al., 2013; Hanauer et al., 2017; Hanauer et al., 2022; Rodenbusch et al., 2016). iRECs aim to be inclusive by supporting all STEM faculty as they engage their students in research, irrespective of their prior research experiences or institutional research capacities. Through iRECs, STEM faculty are provided training to learn about and implement course-based research projects; they are also provided sustained implementation support through the provision of curated instructional resources (e.g., compendium of protocols), technical services (e.g., sequencing of phages genomes, development and curation of databases), and subject matter expertise (e.g., phage biologists and education assessment researchers). Faculty in an iREC implement course-based research projects iteratively, year after year, and are convened regularly as a community to share and advance their research and strategies for mentoring students. Examples of iRECs include Science Education Alliance (SEA), Genomics Education Partnership, and Tiny Earth, each coordinating their own community of hundreds of faculty who implement common course-based research projects (Jordan et al., 2014; Hanauer et al., 2017; Schaffer et al., 2010; Elgin et al., 2017; Hurley et al., 2021).

SEA was established in 2008 with its first cohort of STEM faculty from 13 institutions. In the 2023–2024 academic year, the SEA community consisted of faculty from 16 cohorts and across 145 institutions who collectively engaged over 6,000 primarily freshmen and sophomore students in course-based research. Previously, the effectiveness of SEA was evaluated by assessing outcomes for students participating in the Phage Hunters Advancing Genomic Evolutionary Science (PHAGES) course-based research project, commonly referred to as SEA-PHAGES. A detailed pathway model was first constructed to visualize the student experience, which subsequently led to the development and validation of the Persistence in the Sciences (PITS) assessment tool (Hanauer et al., 2016; Reeves et al., 2020). This combination of large-scale modeling and validated instrument development was then used to empirically demonstrate positive outcomes for students related to their intent to persist in the sciences (Hanauer et al., 2017). Based on these measures of student outcomes, SEA is considered effective at facilitating faculty to implement updated curricular content and pedagogy successfully.

The aim of the present study is to explicate the process through which SEA, and by extension, an iREC, facilitates such pedagogical advancement. To accomplish this, a range of stakeholders in the SEA program engaged in a 2-year study to describe the process through which SEA supports cohorts of STEM faculty (hereafter referred to as SEA faculty) to learn and successfully implement two course-based research projects – the PHAGES project and the Gene-function Exploration by a Network of Emerging Scientists (GENES) project – in place of traditional laboratory courses. Through the development of a detailed pathway model of faculty experiences in SEA, we identified four categories of outcomes that faculty realize through their participation in the iREC, and we describe SEA programming that facilitates the development of those outcomes. These efforts revealed SEA to 1) support faculty to adopt common curricular content (i.e., the PHAGES or GENES projects) and pedagogy (i.e., course-based research) in the context of a collaborative community and 2) support the community to leverage data and one another to iteratively advance their implementation, in a manner that they find professionally rewarding. The sections that follow provide a detailed description of these faculty outcomes and SEA programming, which can serve as a framework to facilitate broader adoption of course-based research and to inform efforts to transform undergraduate science education, more broadly.

## Methods

### OVERVIEW

Pathway modeling is an approach to understand how a program operates through the representation of short-, medium-, and long-term program outcomes (Urban & Trochim, 2009). Usually developed and used by program evaluators, a pathway model shows the theoretical connections between program activities and their intended outcomes, which can give program staff and stakeholders the ability to better “see” how their program is believed to work (Reeves et al., 2020). While pathway models tend to appear visually complex (i.e., spaghetti-like), this complexity better represents the reality of how large-scale programs like an iREC operate in actuality. The SEA faculty pathway model developed herein is intended to reveal the underpinning features of the iREC that support cohorts of faculty to learn and continually advance their implementation of course-based research. The iREC faculty pathway model can also serve as a working framework for initiatives aimed at facilitating pedagogical transformations more broadly. What follows is a description of how we carried out the SEA faculty pathway model creation, refinement, community checking, and interpretation, which involved three types of stakeholder groups: internal SEA researcher group (n=4), small SEA faculty stakeholder group (n=5), and large SEA faculty stakeholder group (n=109).

### Pathway Model Creation by Internal SEA Researcher Group (n=4): Participants and Procedures.

The process of drafting an initial pathway model of the SEA faculty experience began with four individuals: one HHMI SEA program staff member who is familiar with all aspects of SEA programming (Sivanathan); one SEA faculty member who has participated in SEA for over a decade and supported the development and implementation of SEA programming (Monti); and two external evaluators (Gill and Graham) including one (Graham) who had previously contributed to the SEA-PHAGES student pathway modeling project (Hanauer et al., 2017). The team of four met once a week for eight weeks to draft an initial pathway model based on their collective assumptions of how faculty experience the SEA program. This team developed a list of outcomes that SEA faculty experience and realize as part of their engagement with the program. Using a program evaluation and planning tool (The Netway; www.evaluationnetway.com), these outcomes were then organized as short-, medium-, and long-term outcomes based on when these outcomes are likely to be experienced by SEA faculty. Outcomes were also functionally linked to one, that is two outcomes are linked, directionally, when the realization of one outcome is dependent on the other. Activities and programming that supported the development of various outcomes were also identified. The result was a draft pathway model to represent the SEA faculty experience. The model was not intended to capture institutional particularities of the SEA faculty experience but rather to capture an experience that would be typical of most SEA instructors; it was understood that some components of the model were more implicit components of the faculty iREC experience than others. For example, while long-term outcomes such as “Faculty contributions support their tenure and promotion” and “Faculty secure internal and/or external funding” are desired by SEA program staff and faculty alike, this may be mostly aspirational in practice.

### Pathway Model Refinement by Small SEA Faculty Stakeholder Group (n=5): Participants and Procedures.

The aim of this stage was to evaluate, modify, and content validate the draft pathway model. To do so, five SEA faculty were convened. These five faculty are considered expert instructors because of their consistently high student outcomes as measured by the PITS assessment tool (Hanauer et al., 2016), and they represent a diversity of institution types and ethnic and gender identities. After being introduced to the draft pathway model by the internal SEA researcher group and given an opportunity to review it, the small group of SEA faculty responded verbally to ten questions (see Table S1). All responses were recorded via Zoom. The first set of questions was designed to prime them to think deeply about their faculty experience (see “*priming*”). The second set of questions was designed to elicit edits to the pathway model (see “*model annotation*”). The third set of questions was designed to capture additional or lingering thoughts about the pathway model and SEA faculty experience. The internal researcher group then used feedback from this small SEA faculty group to update the pathway model.

### Pathway Model Community Checking by Large SEA Faculty Stakeholder Group (n=109): Participants and Procedures.

**Fig.1.**
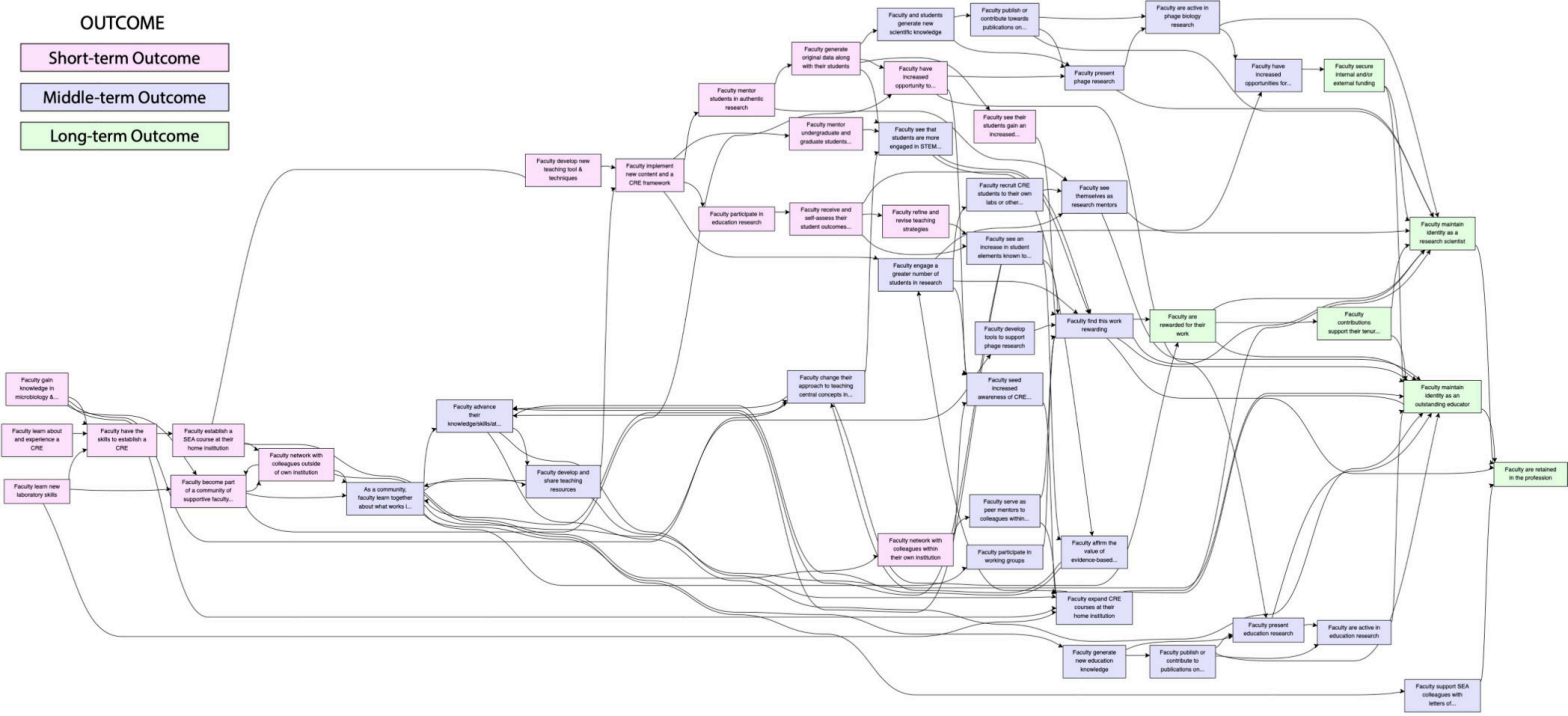
Full SEA faculty pathway model. SEA faculty outcomes occur at different points in time throughout their engagement with SEA: short-term outcomes (pink boxes), medium-term outcomes (lilac boxes), and long-term outcomes (light green). Connections between outcomes are depicted by arrows.

After gathering an initial round of feedback (from the small stakeholder group), data collection efforts were expanded to the broader SEA faculty community. Leveraging the June 2022 Annual SEA faculty meeting, which was hosted virtually, the internal SEA research group presented the pathway modeling process and the pathway model itself to ~150 SEA faculty who attended the meeting. Following the presentation, SEA faculty were placed in breakout groups of ~5 persons and were asked to discuss one aspect of the pathway model. Then, faculty were asked to contribute feedback on that aspect of the model individually via an online survey (Table S2). Following the SEA faculty meeting and our call for feedback, we received 109 responses from SEA faculty at 82 institutions. In general, faculty found the model to be aligned with much of their experience in SEA, as evidenced by comments such as, “Many of the outcomes are reflective of my experiences” and “I definitely resonate with this model.” Therefore, the pathway model, as updated by the small SEA faculty stakeholder group, was found to be representative of the large stakeholder group. A member of the internal SEA researcher group coded the faculty member’s reflections and edited the pathway model to reflect the SEA faculty feedback, resulting in the pathway model presented in this study (Figure 1).

### Pathway Model Interpretation.

**Fig.2.**
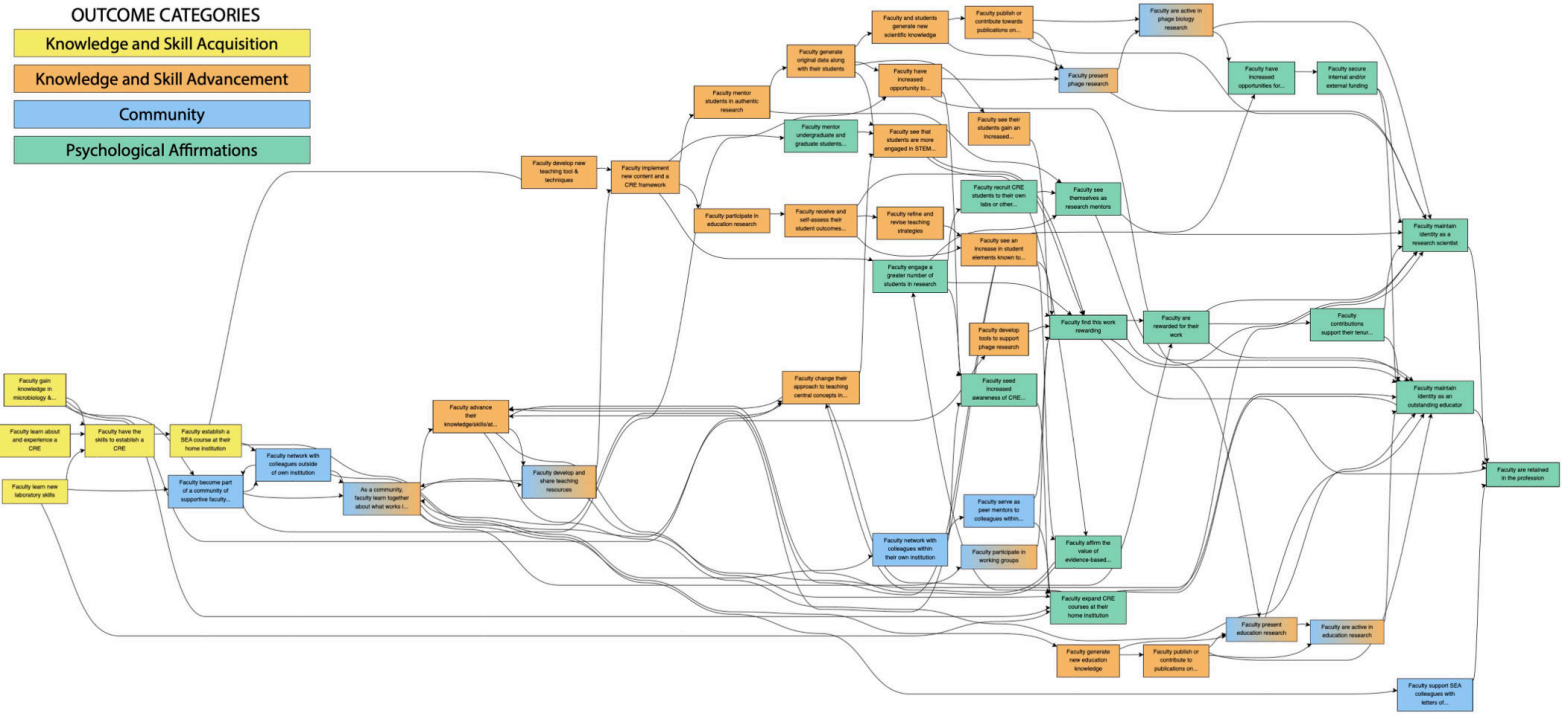
Full SEA faculty pathway model color-coded by outcome category. Outcomes that SEA faculty realize through their participation in the SEA program are grouped into four different categories: Knowledge and Skill Acquisition (yellow boxes), Knowledge and Skill Advancement (orange boxes), Community (blue boxes), and Psychological Affirmation (green boxes). Outcomes that fall into two categories are shaded in both colors.

**Figure 3.**
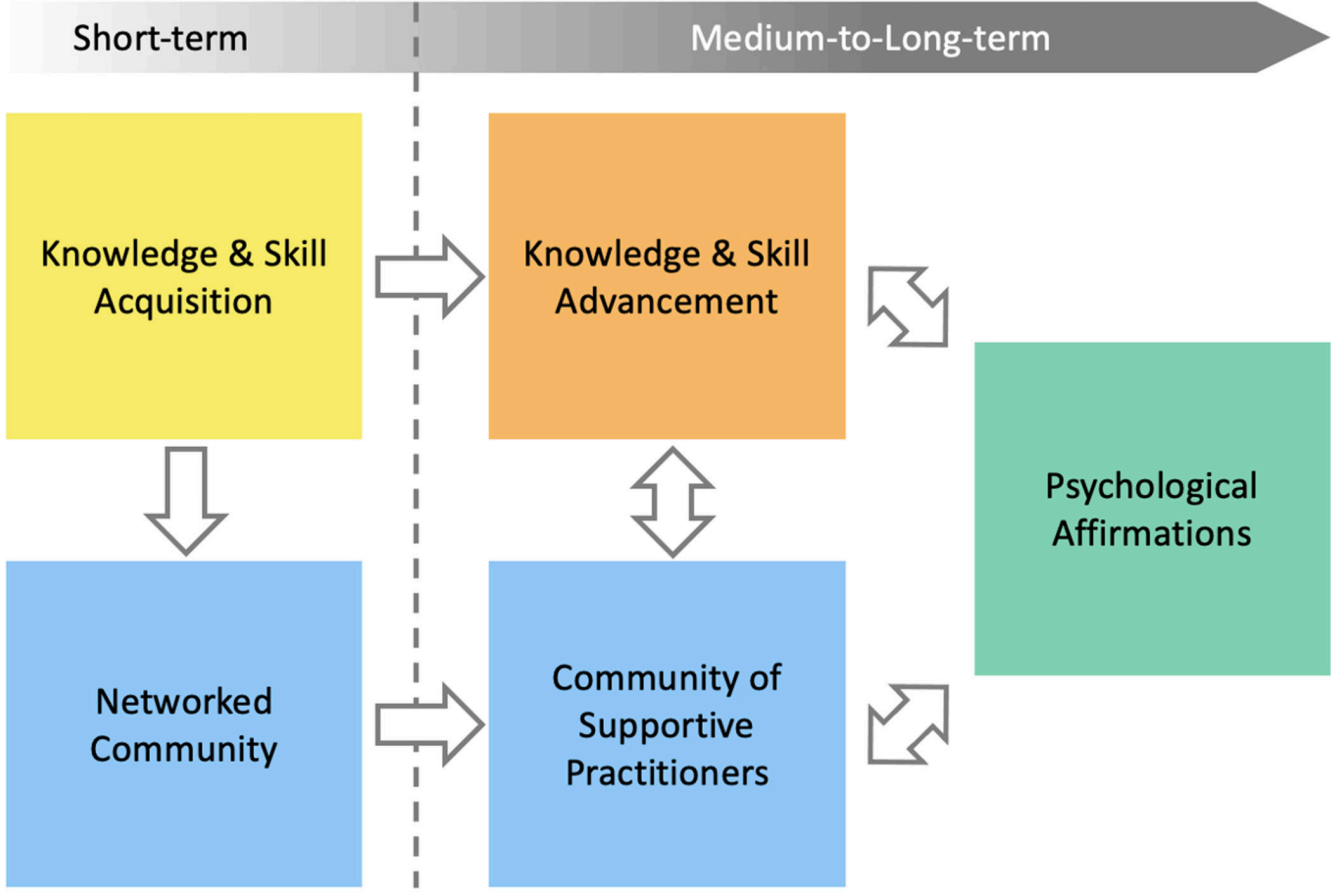
Categorized SEA Faculty Pathway Model. SEA faculty outcomes are grouped into four categories: Knowledge & Skill Acquisition, Knowledge & Skill Advancement, Community, and Psychological Affirmations; the category “Community” can be sub-divided into “Networked Community” and “Community of Supportive Practitioners”. The general relationship of outcomes between these categories, and the timeframe in which faculty are most likely to realize the outcome, are represented by colored boxes and the order in which they appear, from left to right. Within the first year of participating in SEA, faculty realize outcomes related to Knowledge & Skill Acquisition and become networked with other members of the SEA community. In subsequent years, faculty advance their science pedagogy as they engage one another as members of the SEA community. Thus, over time, a community of supportive practitioners grows and learns together. Over time, faculty realize outcomes related to Psychological Affirmations, which promotes their sense of connectedness to their peers, competence as educators, and appreciation for the profession.

Following pathway development, refinement, and community checking as described previously, the internal SEA researcher group analyzed the full pathway model for emergent themes according to guidelines from the systems evaluation protocol (SEP) (Urban et al., 2021). After several rounds of discussion, four major categories of outcomes from the pathway model were identified: Knowledge and Skill Acquisition, Knowledge and Skill Advancement, Community, and Psychological Affirmation. These emergent themes then allowed for the full pathway model – with all its needed complexity – to be depicted and described in a more accessible format by grouping outcomes into these four categories (Figure 2 – full pathway model color-coded by outcome category; Figure 3 – categories pathway model) (Reeves et al., 2020). A final round of feedback on the pathway models, both the full pathway model and the categorized pathway model, was attained. The resulting pathway models and description presented herein are thus representative of the SEA faculty experience from the perspective of SEA program staff, program evaluators, and a majority of SEA faculty.

## RESULTS

### Overview.

To explore the iREC as a model to facilitate the adoption and implementation of new curricular content and pedagogy, we identified outcomes that SEA faculty, in general, realize as a result of their participation in SEA along with activities and programming that support the development of those outcomes (Figure 2 – full pathway model; Figure 3 – categorized pathway model). Broadly, four major categories of faculty outcomes emerged based upon the pathway model interpretation: 1) *Knowledge and Skill Acquisition* (Figure 2 & 3, yellow boxes), which are outcomes related to faculty learning the foundational knowledge and skills needed to initiate a course-based research program at their institution; 2) *Knowledge and Skill Advancement* (Figure 2 & 3, orange boxes), which are outcomes that are the result of a deepening and broadening of knowledge and skills through practice and experimentation; 3) *Community* (Figure 2 & 3, blue boxes), which are outcomes related to faculty becoming networked with others in SEA and engaging one another as a community of practice; and 4) *Psychological Affirmation* (Figure 2 & 3, green boxes), which are outcomes that motivate faculty to sustain their engagement with SEA. For the most part, outcomes fall into one of these four categories, although there are several outcomes that span the categories of *Knowledge and Skill Advancement* and *Community* and have been assigned and color-coded as such.

SEA supports faculty for as long as they are engaged in implementing the PHAGES or GENES projects. Accordingly, the various SEA faculty outcomes occur at different points in time throughout their engagement with SEA. In the pathway model, outcomes occur over three timeframes. *Short-term outcome*s are defined as those likely to occur within the first 1 to 2 years of faculty joining the SEA program; these appear towards the left of the pathway. *Medium-term outcomes* are those that begin developing as short-term outcomes are realized, typically when faculty have participated in SEA for an average of 2–5 years; these appear towards the middle of the pathway. *Long-term outcomes* are those that are the culmination of medium-term outcomes and occur through sustained faculty engagement in SEA; these appear towards the right of the pathway.

What follows are detailed descriptions of the various faculty outcomes from the pathway model, along with SEA programming and activities that facilitate the development of the outcomes. These are presented first by outcome category and timing and then by the relationship of outcomes to one another.

### Outcome Categories & Timing

#### Outcome Category 1: Knowledge and Skill Acquisition

##### Overview:

Outcomes categorized as “Knowledge and Skill Acquisition” relate to SEA faculty learning the foundational knowledge and skills needed to implement the PHAGES or GENES course-based research projects. Course-based research is a relatively new form of teaching for most faculty, and many have not engaged in virus-host research related to the PHAGES or GENES projects. Therefore, a key and initial aim of SEA is to support faculty in acquiring the knowledge and skills necessary to lead a PHAGES or GENES course-based research program at their home institution.

##### Outcomes:

Knowledge and skill acquisition outcomes (Table 1) include faculty learning key concepts and techniques relevant to the PHAGES or GENES course-based research projects. Examples include background information and techniques in microbiology, molecular biology, microscopy, and bioinformatics. Faculty also learn effective approaches for course-based research implementation.

##### Programming & Activities:

Knowledge and skill acquisition outcomes directly result from knowledge-transfer and skill-development activities organized by the SEA program staff. These occur principally through training workshops offered to SEA faculty before their first implementation of the course-based research projects. The workshops are designed for experiential learning, where faculty get an immersive experience in course-based research from the perspective of both a student and an instructor. SEA program staff, along with SEA faculty who already have experience implementing the course-based research projects, serve as instructors for the training workshops and provide insight into practical research activities such as reagent preparation and overseeing the implementation of complex protocols. The training workshops also support faculty in understanding and navigating novel challenges associated with course-based research, such as strategies for managing the uncertainties related to timing and outcomes typical of authentic research. Within a relatively short period of time (i.e., one week of in-person training), SEA faculty are prepared to establish and begin implementing the PHAGES or GENES course-based research projects at their institution.

##### Timing:

Knowledge and skill acquisition outcomes are short-term outcomes, occurring within the first year of faculty joining the SEA program.

### Outcome Category 2: Knowledge and Skill Advancement

#### Overview.

**Figure 4.**
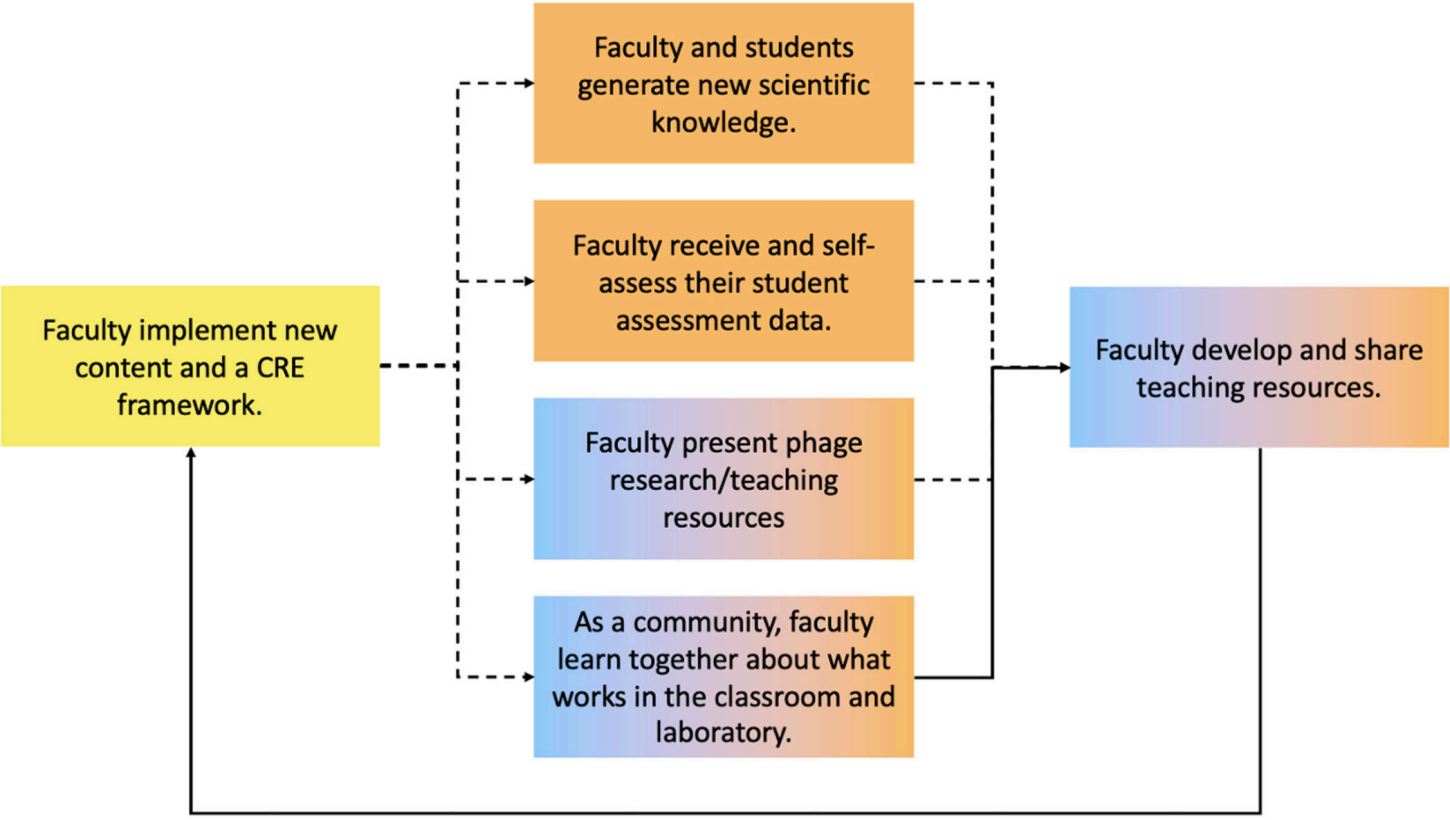
Knowledge and skill advancement through iterative cycles of instruction, feedback and assessment, and experimentation. SEA faculty spend a year engaging a cohort of students in course-based research. Towards the conclusion of each year, faculty receive student assessment data and have opportunities to share and discuss their research findings and teaching strategies with the SEA community. Through these opportunities, faculty learn how to better implement course-based research, including the development of new science pedagogy resources that are shared with community. This is an iterative process. Dashed lines represent outcomes that are indirectly linked in the full pathway model; solid lines represent outcomes that are directly linked.

Outcomes categorized as “knowledge and skill advancement” relate to a deepening and broadening of faculty knowledge and skills for leading PHAGES and GENES course-based research projects through long-term practice and collaboration coupled with experimentation and assessment. As faculty implement the PHAGES and GENES projects at their institutions iteratively, year after year, they leverage their individual experience as well as the collective experience and expertise of members of the iREC (e.g., other SEA faculty, subject-matter experts, and SEA program staff) to advance their scientific and pedagogical knowledge and skills (Figure 4). As a result, they become increasingly more effective at managing the complexities of mentoring a cohort of students in authentic research and are able to continually enhance the research and learning experiences they afford their students.

#### Outcomes.

Outcomes related to faculty advancing their knowledge and skill (Table 2) are numerous and include SEA faculty doing the following: 1) implementing and leading course-based research at their home institutions; 2) mentoring students and monitoring student outcomes (e.g., “faculty and students generate new scientific knowledge” and “faculty receive and self-assess their student assessment data”); 3) communicating and receiving feedback from others in SEA (e.g., “faculty present phage research”); 4) developing and sharing resources and strategies to enhance their course-based research program (e.g., “faculty develop and share teaching resources” and “as a community, faculty learn together about what works in the classroom and laboratory”); and, 5) engaging in research related to their teaching (e.g., “ faculty are active in SEA community education models”; Table 2).

#### Programming & Activities.

Programming and activities that allow faculty to leverage the community to advance their knowledge and skills include early-semester check-ins with program staff and multiple avenues for information sharing and feedback across the community, including curated online community forums, monthly and annual faculty meetings, research symposia, and platforms to support short- and long-term collaborative endeavors. For example, a small group of SEA faculty was supported to collaborate over a 4-week period in the summer of 2020 to develop a resource to guide students in drafting a short-format manuscript describing the research findings from the year-long PHAGES research project. This resource was then shared with the entire SEA community and has been updated annually based on community feedback. In 2022, version 3 of the resource was used by over 40 SEA faculty to support their students in drafting manuscripts of their PHAGES research findings, which were then successfully published in a peer-reviewed journal (Diaz et al., 2020; https://seaphages.org/publications/). In 2023, version 4 of the resource was published with a validated grading rubric based on feedback from over 100 SEA faculty.

Each semester, SEA conducts systematic program-wide student assessment to measure psychosocial variables correlated with student persistence in the sciences (PITS) (Hanauer, 2016). These assessment data are provided to faculty for each section of their students with comparisons to program-wide means allowing for evaluation of their pedagogical approach and advancement over time. Moreover, faculty are invited to participate in phage and education research projects organized by SEA that aim to advance our collective understanding of phage biology and course-based research pedagogy. Examples include manuscripts describing the genetic diversity and inter-relatedness of large collections of phages, as well as the instructional and assessment practices of course-based research, each co-authored by over 100 SEA faculty (Pope et al., 2015; Jacobs-Sera et al., 2020; Hanauer et al., 2022, Hanauer et al., 2023).

#### Timing.

Some outcomes related to knowledge and skill advancement occur as short-term outcomes in the pathway model, but most are medium-term outcomes that are realized after faculty begin to engage with one another as a community of practitioners.

### Outcome Category 3: Community

#### Overview.

Outcomes categorized as “community” relate to SEA faculty becoming organized as a community of practice and collaborating with one another. These outcomes are inherent to SEA as a program designed to support faculty engaged in collaborative course-based research.

#### Outcomes.

Outcomes here include faculty expanding their professional network to include SEA colleagues in addition to other individuals at their own institutions and subsequently engaging with their expanded network as a community of practice. Outcomes for the latter include faculty engaging one another to discuss research and pedagogy (e.g., presenting research data and pedagogical practices at meetings and symposia), co-developing new resources to support their implementation of course-based research (e.g., teaching resources, software supporting genome annotation and comparative bioinformatics), and collaborating to advance STEM pedagogy (e.g., studying key elements of instruction and assessment in course-based research). In addition, outcomes here include faculty supporting each other’s career advancement (Table 3).

#### Programming and Activities.

Specific programming to facilitate networking begins as soon as faculty join SEA. For example, a cohort model is implemented for new faculty who join SEA, with onboarding and training occurring as group activities. Additionally, during the onboarding process, new faculty are paired with experienced faculty, who serve as additional points of contact and “buddies” to help new faculty navigate the program and meetings early in their engagement with SEA. Similarly, at the initial faculty training workshops, a group of experienced SEA faculty serves as facilitators. They continue to serve as a resource for new faculty, particularly as new faculty plan and implement their first iteration of the PHAGES or GENES projects. To support SEA faculty operating as a community of practice, SEA program staff facilitate cross-institutional faculty communications and group work through curated online forums and platforms, as well as annual in-person meetings that provide protected time for faculty to advance their knowledge and skills in phage research and STEM pedagogy. Using SEA infrastructure, faculty share teaching tips and resources with one another, respond to each other’s queries regarding course-based research implementation, and provide feedback on research findings. Some outcomes in this category, such as faculty supporting the professional advancement of one another by serving as peer mentors and writing letters for promotion and tenure, have developed organically within the community without direct SEA programming. Subsequently, program staff began developing infrastructure to facilitate these outcomes.

#### Timing.

Faculty networks develop early as faculty join the SEA program, while outcomes related to SEA faculty operating as a community of practice appear early but are mostly medium-term outcomes that faculty realize when they implement the PHAGES or GENES course-based research projects iteratively, year after year.

### Outcome Category 4: Psychological Affirmation

#### Outcome Category Overview.

“Psychological affirmation” is a multifaceted outcome category that we define as factors that mostly occur external to, but as a result of, faculty engagement with SEA programming. Importantly, these outcomes affirm a faculty’s sense of connectedness to their peers, competence as educators and researchers, and appreciation for the profession. Collectively, and consequently, these outcomes motivate and sustain faculty engagement with SEA, influence their persistence in implementing course-based research, and ultimately impact their identities as STEM faculty – both as researchers and science educators – and their retention in the profession (van Lankveld et al., 2016; Hanauer et al, 2024)

#### Outcomes & Timing.

Psychological affirmation outcomes occur throughout the pathway model and develop over time. Faculty realize short-term psychological affirmation outcomes when they mentor additional students in relation to the PHAGES and GENES course-based research projects. The example here is “faculty mentor undergraduate and graduate students serving as teaching assistants.” Faculty realize medium-term psychological affirmation outcomes when their role as a researcher and research mentor expands beyond the SEA courses. Examples here include “faculty recruit CRE students to their own labs or other research labs” and “faculty have increased opportunities for grants.” Long-term outcomes are realized when faculty are recognized for their contributions to advancing science, science education, and student learning, for example, “faculty contributions support their promotion and tenure,” “faculty maintain identity as an outstanding educator,” and “faculty maintain identity as a research scientist.” SEA facilitates the recognition of faculty contributions by making education and research resources developed by faculty citable and through authorship on publications that are the result of collaborations (https://seaphages.org/publications/; Diaz et al., 2020).

### Relationship of Pathway Model Outcomes

The SEA faculty pathway model depicts the complex ways in which faculty outcomes are interconnected and facilitate the development of one another over time. Thus, beyond the identification of emergent themes of faculty outcomes, an understanding of the relationship between outcomes is important for considering how the various faculty outcomes emerge. The interrelatedness of outcomes is exemplified with a few specific examples.

#### “Knowledge and Skill Acquisition” and “Knowledge and Skill Advancement”

The relationship between the acquisition and the advancement of knowledge and skills is readily observable in the pathway model, with the former necessary for the latter. The underlying logic is that faculty must first have acquired the knowledge and skills to implement course-based research before they are able to learn from and deepen their understanding for doing so. For example, the outcomes “faculty learn about and experience a CRE” and “faculty have the skills to establish a CRE” are prerequisites to outcomes like “faculty receive and self-assess their student outcome data” and “faculty revise and refine their teaching strategies.”

#### “Knowledge and Skill Acquisition” and “Community”

In the SEA, knowledge and skill acquisition occurs in the context of a community. As described previously, faculty training follows a cohort model. Training is supported by various members of SEA, including program staff, experienced SEA faculty, and subject matter experts, who each bring a diversity of experiences and expertise that support faculty learning how to implement course-based research. Consequently, and concomitant to faculty acquiring the knowledge and skills necessary for leading PHAGES or GENES course-based research projects, SEA faculty expand their professional network to include other members of the SEA community. For example, the outcomes “faculty learn new laboratory skills” and “faculty gain knowledge in microbiology and bioinformatics” are seen in the pathway model as facilitating the outcome “faculty network with colleagues outside of their own institution” (Figure 2).

#### “Knowledge and Skill Advancement” and “Community”

Outcomes in these two categories are highly related, highlighted by multiple outcomes being assigned to both categories. For example, “faculty participate in working groups,” “as a community faculty learn what works in the lab and classroom,” and “faculty develop and share teaching resources,” are listed as outcomes in both categories. This inter-relatedness highlights how knowledge and skill advancement for SEA faculty is facilitated by their engagement with one another as a community of practice. This is a reciprocal relationship, with much of community engagement occurring in the context of, and driven by, collaborative efforts to advance their PHAGES and GENES research, course-based research pedagogy, and student outcomes.

#### “Psychological Affirmations” with “Community” and “Knowledge and Skill Advancement”

As described previously, psychological affirmations are outcomes that motivate and sustain faculty engagement with SEA by reaffirming their connectedness to their peers (“community”) and their competence as researchers and science educators (“knowledge and skill advancement”). For the former, the outcome “faculty find this work rewarding” stems from outcomes such as “faculty serve as peer mentors to colleagues within the SEA and to colleagues at their own institution” and “faculty participate in working groups,” which exemplify faculty engaging their peers as part of a supportive community. For the latter, the outcomes “faculty expand CRE courses at home institutions” and “faculty seed increased awareness of course-based research at their home institutions” are a result of faculty being skilled as course-based research instructors.

#### Highly Connected Outcomes (hubs)

In examining the interconnectedness of the various outcomes at a more granular level, we identified two outcomes in the pathway model that are highly interconnected with other outcomes. In pathway modeling, such interconnected elements are known as “hubs” (Urban and Trochim, 2009). A hub may be an important outcome because it represents a highly cumulative outcome (i.e., many other outcomes contribute towards the development of the hub outcome), or the hub is an important stepping stone for the development of multiple other outcomes or both. We identified two highly interconnected hubs in the model.

**Figure 5.**
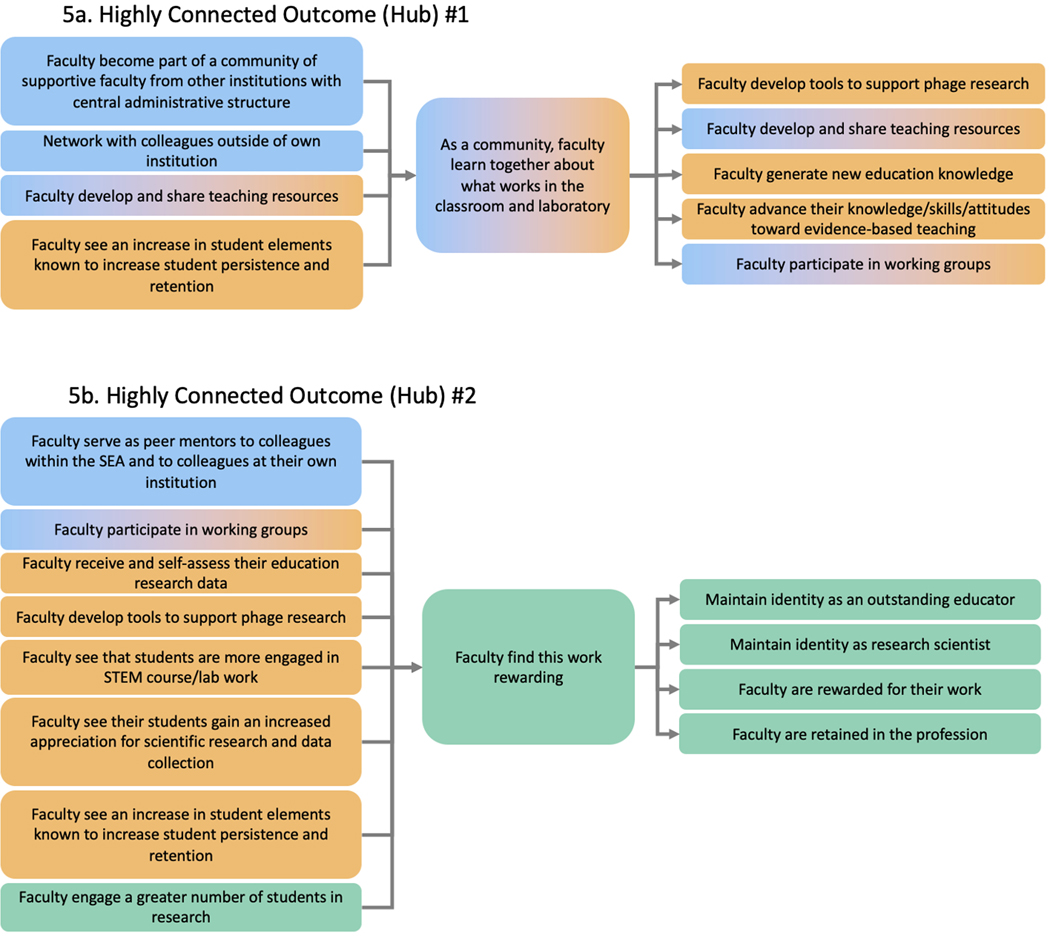
Highly connected outcomes (hubs). Hubs are highly interconnected outcomes that are impacted by and impact multiple outcomes in a pathway model. (A) “As a community, faculty learn together about what works in the classroom and laboratory” was identified as a hub. It is an outcome in the categories of “Knowledge & Skill Advancement” and “Community” and is connected to many outcomes across these two categories. (B) “Faculty find this work rewarding” was identified as a hub. It is an outcome in the category “Psychological Affirmations” and is connected to multiple outcomes across multiple categories.

The first hub identified is “as a community, faculty learn what works in the classroom and laboratory.” This hub is an outcome in the categories of “Knowledge and Skill Advancement” and “Community.” As shown in Figure 5a, this hub is central to multiple other outcomes in the same categories. Additionally, one of these supported outcomes – “faculty develop and share teaching resources” – also feeds into this hub, thus creating a positive feedback loop within these categories. Collectively, this hub highlights that a central outcome for SEA faculty is engagement in a community of practice that supports and advances their knowledge and skills in implementing course-based research.

A second hub, “faculty find this work rewarding,” is the most highly connected outcome in the SEA pathway model (Figure 5b). This outcome underscores other outcomes in the model that contribute to faculty having a positive connection to SEA, including being able to engage and support one another professionally as a community of practice and observing positive outcomes for their students that result from their instruction and mentorship. This hub, in turn, supports multiple psychological affirmation outcomes. It depicts that when faculty find the work rewarding, it bolsters their professional identities and retention in SEA and the profession (Hanauer et al, 2024).

#### Summary of Pathway Model Outcomes

The SEA faculty pathway model is a visual and chronological representation of the large-scale and complex process through which an iREC facilitates the adoption and continual advancement of course-based research pedagogy. As can be tracked through the SEA pathway model (Figure 3), faculty first acquire the knowledge and skills to implement the PHAGES or GENES research projects. Simultaneously, faculty become networked with a community that includes other faculty (new and experienced SEA faculty), subject matter experts (i.e., active researchers of phage biology, assessment, and program evaluation), and SEA program staff. As SEA faculty implement course-based research at their institution, they leverage the varying experiences and expertise of the SEA network to continually enhance their course-based research program. SEA faculty do so by engaging the SEA network in dialogue about their course outcomes, working collaboratively to develop and experiment with new instructional tools and strategies, and participating in education research. By doing so iteratively, year after year, in the context of a community of practice, SEA faculty have opportunities to advance their knowledge and skills for implementing course-based research. Towards the later stages of the pathway model, it is observed that the opportunity to engage with others as a supportive community of practice, to facilitate positive outcomes for their students, and to advance both science and science education reinforces their identity as STEM faculty – both as researchers and science educators – and creates additional opportunities related to their career advancement. These outcomes ultimately contribute to their retention in the profession.

## Discussion

There are many initiatives designed to facilitate pedagogical transformations in undergraduate STEM education. Most are successful at supporting faculty in learning why and how to implement evidence-based teaching practices. However, these initiatives less often facilitate the skill refinement needed for true transformation in teaching (e.g., Bathgate et al., 2019; Ebert-May et al., 2011; Stains et al., 2014). Researchers have pointed to a need for more “opportunities for teachers to collaborate with colleagues and other experts to improve their practice” as well as for science educator training to include opportunities for reflection and iteration while being guided by student learning data (Loucks-Horsley, 1998); there are also calls to move away from episodic training opportunities to a model that supports continual professional learning (Webster-Wright, 2009). Taken together, the establishment of long-term communities of practice that position educators to leverage data and evidence to continuously improve their teaching is needed. These recommendations are echoed by the National Science and Teaching Association (NSTA Board of Directors, 2016). Many existing initiatives have indeed established communities of practice. Defined as communities in which members are connected and share common values, purpose, and resources (Allee, 2007), these communities of practice, however, generally disband after 1 – 4 semesters (Beach et al., 2016) and do not necessarily have data on student learning to inform their practice (Ebert-May et al., 2011; Stains et al., 2014).

As described in the summary of the results presented in this study, STEM faculty in an iREC are organized as a community of practice who additionally leverage both data and the collective experiences and expertise of the community to engage in sustained advancement of their teaching. For example, faculty who join the SEA program are first supported to learn how to implement course-based research while also being networked with peer practitioners, experts, and program staff. These are short-term outcomes for SEA faculty and are likely similar to outcomes for faculty engaged in the majority of faculty training initiatives. Importantly, a community of practice in which faculty are engaged in collaborative and iterative advancement of their teaching only emerges later in the SEA faculty pathway model as mid- to long-term outcomes. The SEA faculty pathway model suggests four interrelated and underpinning features of an iREC that facilitate the development and functioning of such a community of practice:

First, an iREC is focused on common content. This is in addition to common values, purpose, and resources typical of communities of practice. In the SEA program, the focus is on the PHAGES and GENES course-based research projects. This commonality of content allows for the diversity of teaching approaches, experiences, and expertise represented in the community to be leveraged by faculty to inform and advance their own teaching. Additionally, common content facilitates the development and use of standardized evaluation tools that make data directly comparable by faculty for the purposes of gauging the effectiveness of their teaching strategies.

Second, embedded within this community are a range of subject-matter experts, from phage biology researchers to assessment and program evaluation researchers, who are invested in and collaborate to advance science pedagogy. Designing and executing assessments, evaluating that data, and leveraging that data to advance research and teaching practices is a skilled and time-consuming undertaking. By providing faculty with comprehensive data translations and data summaries, for example, about their teaching, faculty are then better positioned to improve their practice in a data-driven manner.

Third is the long-standing nature of an iREC community. The advancement of any practice is an iterative process, and the stability of an iREC allows faculty to remain engaged with the community to build their expertise and advance their teaching gradually and, importantly, continually. With an ever-evolving education landscape, the opportunity for continual advancement of teaching skills and expertise is important if faculty are to meet the evolving needs of their student populations and the demands of the times.

Fourth, the intentional positioning of an iREC as a professional community is designed to support all its members in a collective and collaborative endeavor that additionally recognizes and credits their contributions. Faculty thus feel connected to one another and are committed to each other’s success, supporting one another in overcoming difficulties and in advancing their course-based research implementation. As described in the results section of this study, the opportunity for faculty to engage with one another in this way is rewarding and serves to reinforce and sustain their engagement in this community of practice.

The iREC features described in this study go beyond facilitating skilled implementation of new curricula content and pedagogy. Because the iREC is a large, diverse, and long-standing community of faculty and subject matter experts, it offers a novel and unique lens to explore complex questions that may have implications for science educators and science education more broadly. Prime examples include recent studies that described course-based research pedagogy through an examination of the instructional and assessment goals and practices of over 100 SEA faculty (Hanauer et al., 2022, Hanauer et al., 2023). Such studies elucidate the mechanisms by which course-based research promotes positive outcomes for students and thereby allow for the impactful aspects of this form of teaching to be extrapolated to other areas of science education. Beyond dissemination, an iREC thus offers a strategy also to facilitate the development and evolution of updated curricula content and pedagogy. In this way, an iREC shares similarities with discipline-based education research (DBER), which relies on the combination of expertise from education researchers and perspectives of those who practice in a particular discipline to advance teaching and learning (National Research Council., 2012). In an iREC, a wider field of practitioners – in this case, SEA faculty – are included as collaborators in the research being conducted. Doing so enables STEM faculty in an iREC to both have a bigger voice in shaping science education research questions as well as stay better informed with advances that are relevant to their teaching.

## Conclusion.

The SEA faculty pathway model presented herein describes the iREC as an innovative model for facilitating pedagogical advancement by STEM faculty, both within and beyond their own classrooms. In particular, faculty in an iREC have agency, are not just consumers of education expertise, and find this work professionally rewarding. As a model, the iREC thus offers insight into an approach that can facilitate a transformation in science education at scale by fostering a culture of continuous pedagogical advancement amongst STEM faculty and in a manner that centers the success and advancement of both students and faculty. The SEA program pathway model presented herein is an iREC exemplar because it lays out the components, stages, and relationships that support such a community of STEM faculty. We invite members of the STEM community to begin exploring the utility of the iREC as a model for enhancing existing efforts and infrastructure to better support STEM faculty in transforming undergraduate science education.

## Supplementary Material

Supplementary Tables S1 - S2

## Figures and Tables

**Table 1:** Knowledge and Skill Acquisition Outcomes

SHORT-TERM OUTCOMES
Faculty learn new laboratory skills
Faculty gain knowledge in microbiology & bioinformatics
Faculty learn about and experience a CRE
Faculty have the skills to establish a CRE
Faculty establish a SEA course at their home institution
MID-TERM OUTCOMES
none identified
LONG-TERM OUTCOMES
none identified

**Table 2:** Knowledge and Skill Advancement Outcomes

SHORT-TERM OUTCOMES
Faculty implement new content and a CRE framework
Faculty generate original data along with their students
Faculty see their students gain an increased appreciation for scientific research and data collection
Faculty develop new teaching tool/techniques
Faculty refine/revise teaching strategies
Faculty receive and self-assess their education research data
Faculty mentor students in authentic research
MID-TERM OUTCOMES
Faculty and students generate new scientific knowledge
Faculty present phage research
Faculty publish science findings (GenBank, MRA)
Faculty are active in phage biology research
Faculty develop tools to support phage research
Faculty change their approach to teaching central concepts in biology
Faculty generate new education knowledge
Faculty present education research
Publish STEM education findings
Faculty are active in SEA community education research
Faculty advance their knowledge and skills/attitudes toward evidence-based teaching
Faculty develop and share teaching resources
Faculty see that students are more engaged in STEM course/lab work
Faculty see an increase in student elements known to increase student persistence and retention
Faculty participate in working groups
As a community, faculty learn together about what works in the classroom and laboratory
Faculty facilitate or lead expansion of CRE courses at home institution
LONG-TERM OUTCOMES
none identified

**Table 3:** Community Outcomes

SHORT-TERM OUTCOMES
Faculty network with colleagues outside of own institution
Faculty become part of a community of supportive faculty from other institutions with central administrative structure
Faculty network with colleagues within their own institution
MID-TERM OUTCOMES
Faculty participate in working groups
Faculty are active in SEA community education research
Faculty are active in collaborative phage research
Faculty participate in working groups
As a community, faculty learn together about what works in the classroom and laboratory
Faculty develop and share teaching resources
Faculty present education research
Faculty present phage research
Faculty support SEA colleagues with letters of recommendation
Faculty serve as peer mentors to colleagues within the SEA and to colleagues at their own institution
LONG-TERM OUTCOMES
none identified

**Table 4:** Psychological Affirmation Outcomes

SHORT-TERM OUTCOMES
Faculty mentor undergraduate and graduate students serving as Teaching Assistants
MID-TERM OUTCOMES
Faculty engage a greater number of students in research
Faculty see themselves as research mentors
Faculty recruit CRE students to their own labs or other research labs
Faculty have increased opportunities for grants
Faculty find this work rewarding
Faculty affirm the value of evidence-based teaching
Faculty seed increased awareness of CRE at home institution
LONG-TERM OUTCOMES
Faculty maintain identity as an outstanding educator
Faculty maintain identity as a research scientist
Faculty secure internal and/or external funding
Faculty contributions support their tenure and promotion
Faculty are rewarded for their work
Faculty are retained in the profession
