# Supplementary material for: An inclusive Research Education Community (iREC) Model to Facilitate Undergraduate Science Education Reform
Source: Front Educ (Lausanne). Author manuscript; Available in PMC 2024 Dec 16. (PMC11649309; doi:10.3389/feduc.2024.1442318)
Supplement: Supplementary Tables S1 - S2 [file NIHMS2021733-supplement-Supplementary_Tables_S1_-_S2.pdf]

Table SI. Questions asked to the small stakeholder group during two pathway modeling feedback sessions.

Purpose	Question
Priming	1. What do you see as the main value or contribution of the SEA faculty community for instructors?
Priming	2. How does this depiction (pathway model) of the SEA faculty experience resonate with your own observations?
Model annotation	3. Is anything missing from the model?
Model annotation	4. What do you think are the most important activities in the model?
Model annotation	5. What do you think are the most important outcomes in the model?
Model annotation	6. What do you think are the most important links or connections in the model?
Additional data gathering	7. Think about your SEA experience at your current institution. What is the most important thing someone should know about your institutional context?
Additional data gathering	8. What are key elements of context that influence success or failure of the SEA faculty community?
Additional data gathering	9. Is there anything that you haven't told us yet that would make the model better?
Additional data gathering	10. Do you have any other thoughts that you would like to share related to your experience with the SEA faculty community?

Table S2. Task asked of the large stakeholder group during the Annual SEA meeting.

<table>
<tr><td>1. Take 2 minutes to introduce yourselves</td></tr>
<tr><td>2. Take 2 minutes to read through the pathway below. Look at the outcomes (boxes) and look at how one outcome is linked to the next.

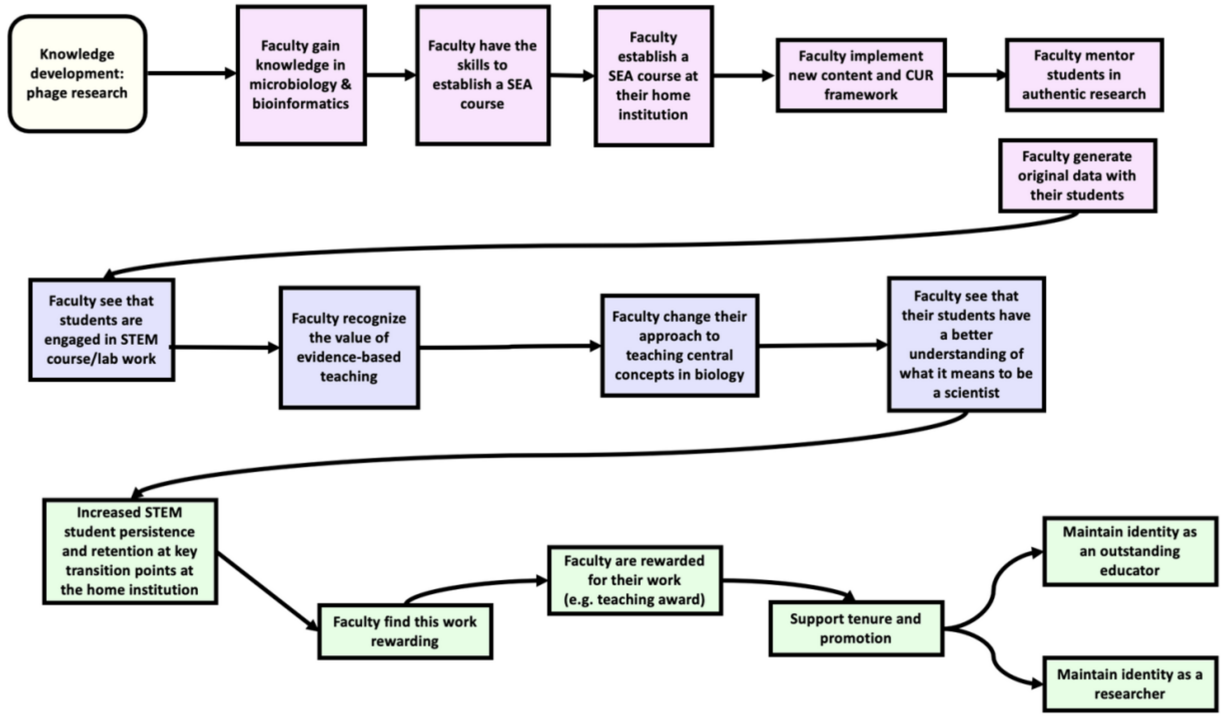

</td></tr>
<tr><td>3. Then take ~ 15 minutes to discuss with your group, the discussion prompts below.

a) Which outcomes in the model are reflective of your experience as a SEA faculty?
b) Are there any outcomes that are aspirational for you or that you would like to experience more of?</td></tr>
<tr><td>4. When prompted, take 5 minutes to provide your reflection on the pathway in the space below. Please write 5-10 sentences reflecting on the SEA Faculty Community Model, focusing on the pathway above.</td></tr>
</table>
